# Immune modulation as a therapeutic strategy in bone regeneration

**DOI:** 10.1186/s40634-014-0017-6

**Published:** 2015-02-07

**Authors:** Claudia Schlundt, Hanna Schell, Stuart B Goodman, Gordana Vunjak-Novakovic, Georg N Duda, Katharina Schmidt-Bleek

**Affiliations:** Julius Wolff Institut and Center for Musculoskeletal Surgery, Charité - Universitätsmedizin Berlin, Augustenburger Platz 1, 13353 Berlin, Germany; Berlin-Brandenburg Center for Regenerative Therapies, Charité – Universitätsmedizin Berlin, Augustenburger Platz 1, 13353 Berlin, Germany; Department of Orthopaedic Surgery and (by courtesy) Bioengineering, Stanford University Medical Center Outpatient Center, 450 Broadway St., M/C 6342, 94063 Redwood City, CA USA; Department of Biomedical Engineering and Department of Medicine, Columbia University, 622 west 168th Street, VC12-234, 10032 New York, NY USA

**Keywords:** Osteoimmunology, Regeneration, Macrophages, Immune modulation, Bone healing, Revascularization

## Abstract

We summarize research approaches and findings on bone healing and regeneration that were presented at a workshop at the 60th annual meeting of the Orthopedic Research Society (ORS) in New Orleans in 2014. The workshop was designed to discuss the role of inflammation in bone regeneration in the context of fundamental biology, and to develop therapeutic strategies that involve immune modulation. Delayed or non-healing of bone is a major clinical problem, with around 10% of fracture patients suffering from unsatisfying healing outcomes. Inflammation is traditionally seen as a defense mechanism, but was recently found essential in supporting and modulating regenerative cascades. In bone healing, macrophages and T- and B-cells interact with progenitor cells, bone forming osteoblasts and remodeling osteoclasts. Among the cells of the innate immunity, macrophages are promising candidates for targets in immune-modulatory interventions that would overcome complications in bone healing and bone-related diseases. Among the cells of the adaptive immune system, CD8+ T cells have been shown to have a negative impact on bone fracture healing outcome, whereas regulatory T cells could be promising candidates that have a positive, modulating effect on bone fracture healing. This workshop addressed recent advances and key challenges in this exciting interdisciplinary research field.

## Introduction

### Bone healing

**Figure 1:**
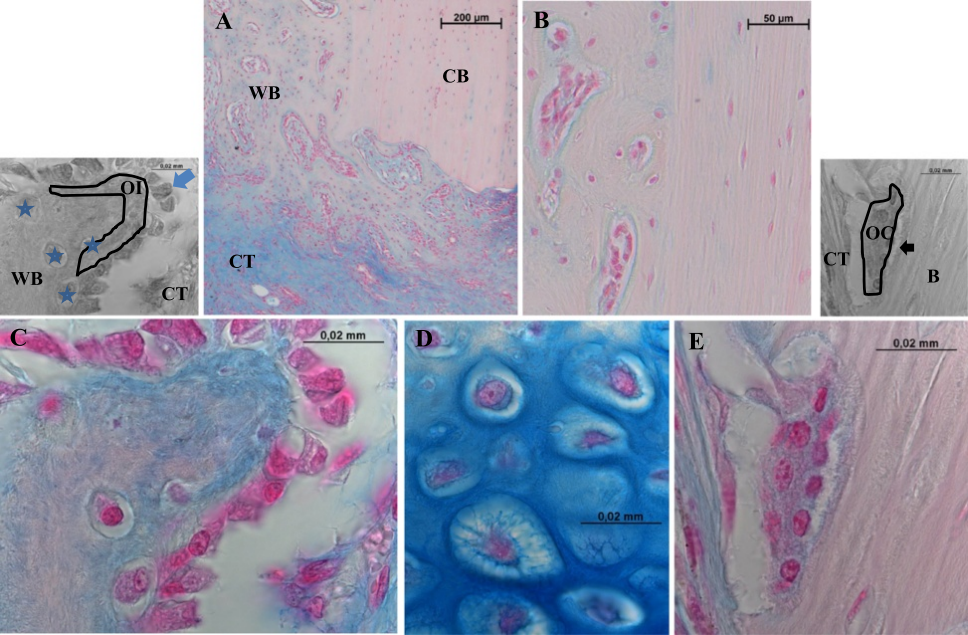
**Bone healing.** A regenerative process leading to bone formation with restored form/ function if successful. **A**: Intramembranous bone formation along osteomized cortical bone (CB) clearly shows the newly formed woven bone (WB) on the left. Further from the bone edge connective tissue is visible, that remains in the osteotomy gap at this healing stage. **B**: The difference between lamellar bone (CB) on the right is clearly visible when compared to the heterogeneous newly formed woven bone (WB) on the left. Important cells in bone formation: **C**: Cells depositing new bone are osteoblasts. The small black and white image explains this image. The arrow indicates an osteoblast in typical palisade form sitting in a row with other osteoblasts on the surface of newly synthesized bone. The region marked with a line and stained blue in “C” shows osteoid (OI), the extracellular matrix, yet unmineralized, synthesized by the osteoblasts. Freshly embedded in the newly formed woven bone at least 4 osteocytes can be seen, surrounded by mineralized matrix (★). **D**: Endochondral bone formation is initiated by a cartilaginous phase, with chondrocytes becoming hypertrophic and then being replaced by osteoblasts and woven bone. **E**: Osteoclasts (OC) are multinuclear bone resorbing cells, sitting on the bone surface. Clearly visible is the ruffled border, which is the actual bone resorbing area of the active osteoclast. (Images are taken from a large animal bone healing model in sheep, 3 mm osteotomy gap, stable external fixation, staining: Alcian blue).

Vertebrates have a bony skeleton that acts as a scaffold for the body. Healthy bone has a remarkable capacity for self-repair (Figure 1). Although bone healing is for the most part an efficient process, around 10% of patients suffer from delayed healing or non-union, leading to the application of complex, expensive, and often invasive, treatment strategies [1].

**Figure 2:**
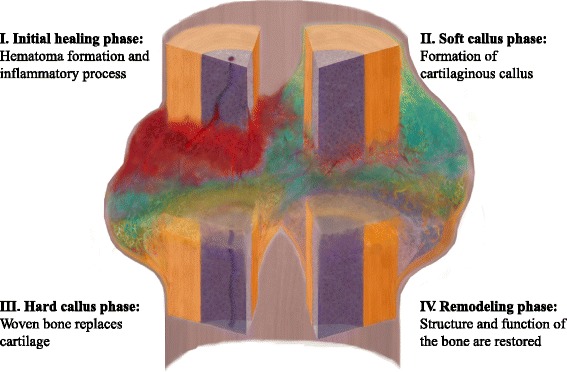
**Different phase of the bone healing process.** This scheme shows the consecutive/partly overlapping bone healing phases (I-IV) occurring in secondary bone healing. The illustration follows the tissue coloring of the histological Movat‘s Pentachrome staining, where bone appears yellow, cartilage appears green, bone marrow is purple, vessels are depicted in red.

Fracture healing is a highly complex process that includes the participation of different cell types (immune cells, progenitor cells, mesenchymal cells) [2] and their signalling molecules (cytokines, growth factors, chemokines) [3]. The healing process, which is initiated after injury, results in effective reconstitution of the initial structure of bone tissue, without scar formation. The regenerative reconstitution can be divided into several consecutive steps: inflammation, soft callus formation, hard callus formation, and remodelling (Figure 2).

**Figure 3:**
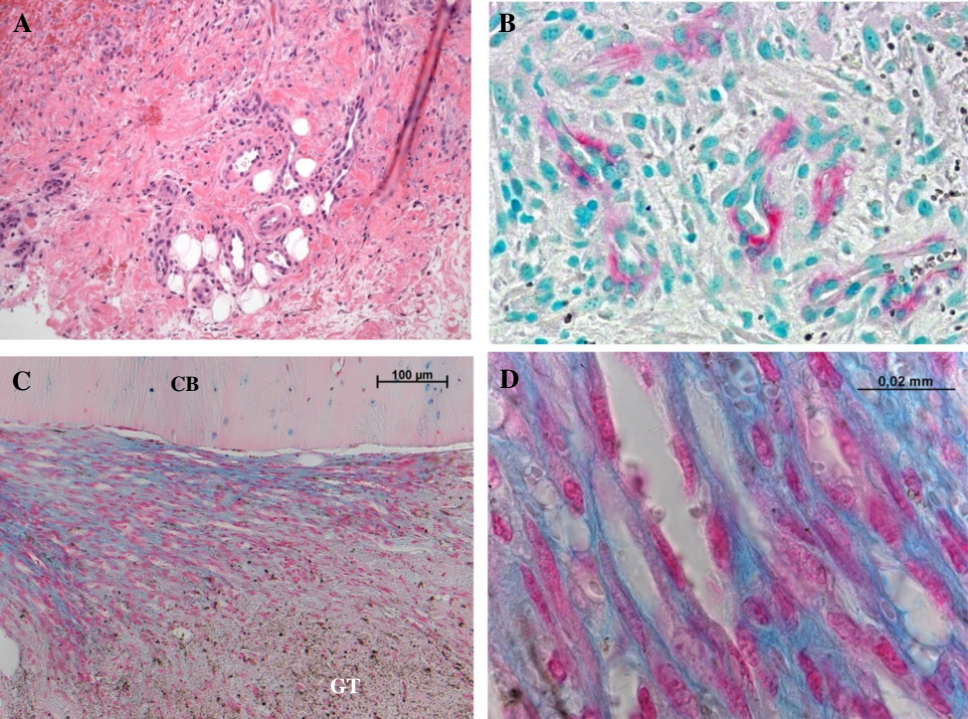
**Onset of revascularization in bone healing.** Seven days after performing a standardized osteotomy in a sheep‘s tibia, the maturating granulation tissue clearly shows newly formed vessels (**A**: hematoxilin-eosin staining; 20 × magnification, **B**: α-SMA, methylgreen counterstaining, vessels depicted in red, 40 × magnification). For these vessels to be this highly developed, revascularization in the hematoma must have started earlier. **C** and **D** (Alcian blue staining): The importance of the revascularization becomes evident when observing the cutting edge of cortical bone (CB) where a high number of vessels developed between bone and granulation tissue remnants (GT). The density of this capillary formation is illustrated in D, where endothelial cells, indicating vessel borders, lie nearly wall to wall.

The inflammatory phase starts with the formation of a hematoma caused by the influx of blood following tissue injury and vascular disruption. Because of its angiogenetic and osteogenetic potential, the formation of fracture hematoma in the early healing phase is an indispensable step for successful healing [4,5]. Removal of the early fracture hematoma was shown to impair bone healing in animal studies, whereas transplantation of the hematoma leads to ectopic bone formation [6,7], demonstrating its osteogenic potential. During the inflammatory phase, the fracture area is characterized by hypoxia (low pH, high lactate) that induces expression of the transcription factor *Hypoxia inducible factor 1α* (Hif1α) that further triggers the expression of angiogenic factors such as *Vascular Endothelial Growth Factor* (VEGF). Revascularization is a prerequisite for the repair phase, including the formation of new cartilage and finally new bone (Figure 3). Immune cells, located in the fracture area, release cytokines as a consequence of an injury [8]. This leads to a recruitment of mesenchymal stromal cells (MSC) to the site of injury where they further proliferate and differentiate thus promoting revascularization.

### Osteoimmunology

**Figure 4:**
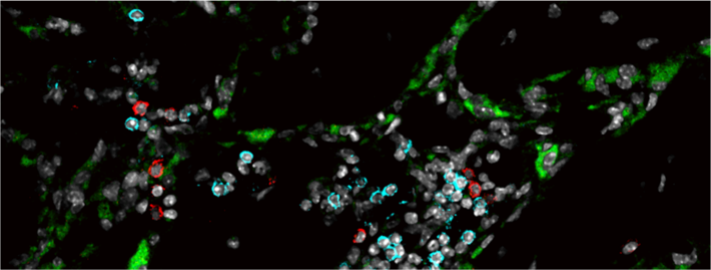
**Interactions between osteoclasts and immune cells in a mouse osteotomy model.** In areas of newly formed woven bone (14 days after fracture, mouse model) the tight interaction between osteoclasts (green (katepsin K)) and immune cells (T cells = red (CD3); B cells = blue (B220)) is eminent. Osteoclasts line the dark areas of newly formed bone in this image.

The immune and skeletal systems share a number of regulatory and signaling molecules. This cross-linkage of the immune and skeleton systems has led to new directions in research and the emergence of “osteoimmunology” [9]. Bone is constantly remodelled due to the action of osteoblast and osteoclast. Osteoblasts are bone-producing cells which are derived from MSCs. Osteoclasts are the bone resorbing counterparts to bone forming osteoblasts. They differentiate from hematopoietic stem cells (HSC) and thus share the same progenitor as cells of the immune system (Figure 4).

In numerous autoimmune diseases, disorders of the skeletal system are commonly found. This has highlighted the fact that there is little known concerning the direct and indirect interactions between bone and the immune system [10-12]. However, recent research clearly showed the influence of the immune system on the bone healing success [13], thus identifying the immune system as a possible tool for new therapeutic approaches. The interconnectivity and complexity of cellular and molecular interactions between the immune system and bone tissue creates a major challenge to develop therapeutic approaches that can specifically target one system without detrimentally affecting the other. With aging of the population in industrialized countries, understanding of this interplay appears to be crucial.

## Review

### Impact of the immune system on bone fracture healing

#### Innate immunity

##### Macrophage

Innate immunity is the first line of defense to recognize and attack pathogens entering the body. This includes physiological barriers (epithelial and mucosa) but also cellular defense mechanisms accomplished by macrophages, mast cells and natural killer cells [14].

**Figure 5:**
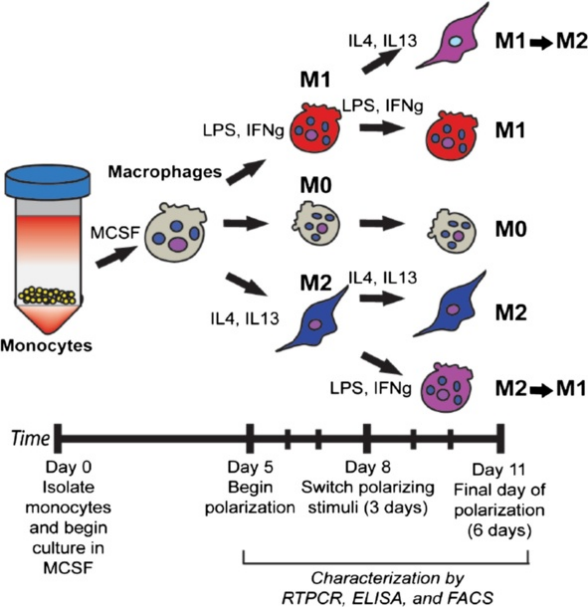
**Derivation of subsets of human macrophages from monocytes.** Monocyte-derived macrophages were exposed to M1- or M2-polarizing stimuli for 3 days followed by polarizing stimuli of the other phenotype for an additional 3 days (M1- > M2 and M2- > M1). Unstimulated macrophages (M0) or macrophages cultured under M1- or M2-polarizing stimuli for 6 days (M1 and M2), with a media change at day 3, served as controls. Reproduced with permission from [8].

The innate immune system plays an important role in tissue repair and in maintaining tissue homeostasis. Among the cells of the innate immunity, tissue resident macrophages are recognized as key elements for the orchestration of the recovery processes to re-establish tissue integrity and function after damage [15]. Macrophages, originally believed to be solely pro-inflammatory and destructive phagocytes, were found in 1992 to have ability to convert to a pro-healing phenotype [16]. Since then, it has been shown that macrophages are necessary for angiogenesis, wound healing, tumor growth, and limb regeneration [17-20]. To distinguish this new phenotype from their familiar “classically activated” counterparts, these macrophages were referred to as “alternatively activated”. Since then, these “M2” macrophages, named following the helper T cell nomenclature (Th1/Th2) and in contrast to pro-inflammatory “M1” macrophages, have been associated with the resolution of wound healing *in vivo* in chronic leg ulcers [21], atherosclerotic lesions [22], traumatic spinal cord injury [23], and inflammatory renal disease [24]. For research studies and therapeutic applications, monocytes isolated from peripheral human blood can be differentiated into macrophages through the addition of the monocyte colony stimulating factor MCSF, and polarized to different macrophage phenotypes via the addition of specific cytokines [25] (Figure 5).

**Figure 6:**
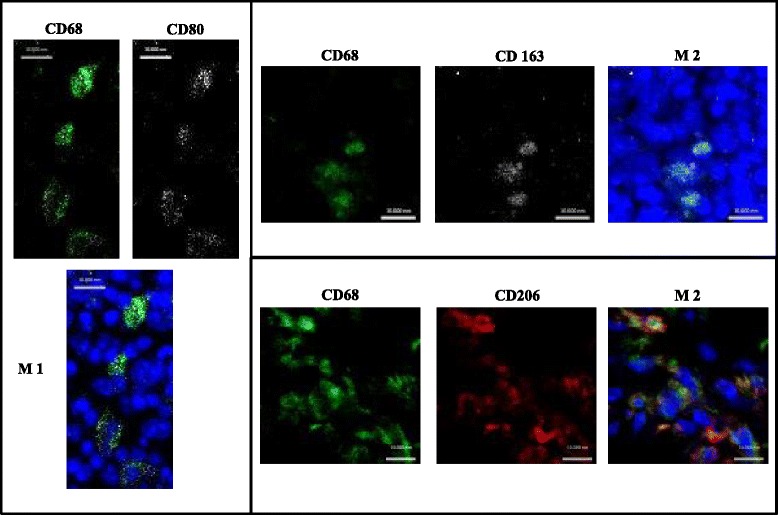
**During bone regeneration different types of macrophages are found in a mouse osteotomy model.** M1 macrophages positive for the CD68 macrophage marker and the M1 marker CD80 are found, for the M2 macrophages we detected cells positive for CD68 and CD163 and other M2 macrophages positive for CD68 and CD 206.

Depending on the micro-environment, macrophages display distinct activation (also called polarization) states. They are divided into the “classical activated” pro-inflammatory M1 type macrophages and into the “alternatively activated” anti-inflammatory, pro-tissue regeneration and repair M2 type macrophages. Within the M2 population, there are different subtypes, characterized by their surface receptor expression and cytokine secretory profile, as: M2a (anti-inflammatory), M2b (immune-regulatory) and M2c (remodelling) [26] (Figure 6).

### Role of macrophages in joint replacement-induced osteolysis: an example of osteoimmunology

Total joint replacement (TJR) is a successful orthopaedic intervention for end stage arthritis. Wear particles are an unwanted by-product of all TJRs and are the cause for the development of chronic inflammation at the implant site [27]. A consistent inflammatory reaction leads to delayed osseointegration of the implant and finally to osteolysis (periprosthetic bone loss) and implant loosening. So far, there are no nonsurgical treatment strategies to overcome wear particle-induced osteolysis. Macrophages are one of the first cells infiltrating the inflammatory reaction induced by wear particles in TJRs [28]. Because of their plasticity, macrophages could be a promising therapeutic target, in the special case of wear particle-induced osteolysis to enhance osseointegration of the implant, and in general to enhance bone healing.

One main key regulator for the migration and infiltration of monocytes and macrophages is the monocyte chemoattractant protein-1 (MCP-1) [29]. Gibon et al. showed in 2012 that a systemic therapeutic intervention in the MCP-1-CCR2 chemokine-receptor axis (CCR2 is the receptor for MCP-1) leads to less systemic migration of macrophages to the site of particle infusion and further, to decreased osteolysis [30,31]. The reverse effect was observed after local administration of MCP-1. This study gave the first hint that the MCP-1-CCR2 axis plays an important role in the recruitment of macrophages to the localization of wear particles. It also showed that bone injury is not only a local event but rather a systemic process affecting further tissues and organs. Blocking of the MCP1-CCR-2 axis by addition of a mutant MCP-1 protein, 7ND, which competes with MCP-1 for the binding to CCR-2 on macrophages, leads also to a decreased migration of macrophages *in vitro. In vivo* studies using 7ND are currently in progress. In the presence of 7ND, a diminished secretion of pro- as well as anti-inflammatory cytokines by macrophages was observed ([32,33]). The simultaneous decrease of pro- and anti-inflammatory cytokine secretion could be explained by a re-establishment of the homeostatic status. Both studies illustrate the crucial role that macrophages play in regulating inflammatory processes in bone probably due to their secreted cytokine profile. An investigation of the responsible macrophage subset would be an interesting and indispensable question to answer with regard to the development of nonsurgical treatment approaches for wear particle-induced osteolysis in humans. Recent *in vitro* and vivo studies have demonstrated that polarization of macrophages from an M1 to an M2 phenotype using local delivery of IL-4 mitigates wear particle-induced bone loss [34].

### Modulation of macrophages in tissue engineering approaches: revascularization of bone grafts

In tissue engineering, revascularization of the transplanted tissue is a crucial prerequisite for a complete integration and functionality of the transplant in the host. Living, functional bone grafts, customized to the patient´s need, are of great clinical need e.g. in the case of congenital abnormalities, cancer resections and trauma [35].

The tissue engineering paradigm involves the use of the patient’s own stem cells capable of osteogenesis and vasculogenesis, biomaterial scaffolds that act as a template for bone formation, and a bioreactor providing nutrition, oxygen supply and regulatory signals. In an ideal case, the missing or defective bone should be repaired using a living, functional bone graft that corresponds to the exact shape of the bone being replaced or regenerated. Current studies are achieving this goal through the use of the patient’s own stem cells and image-based and guided scaffold and bioreactor design. One of the main challenges is to ensure the revascularization with blood perfusion of the bone graft, to maintain its viability and function. The developing vasculature guaranties the needed supply of nutrients and, during normal development, serves as a template for the forming bone. Therefore, for successful bone tissue engineering strategies a synergistic formation of the new bone and new blood vessels is an indispensable condition [36].

Because of their early appearance in inflammatory processes, the action of macrophages and their polarization state could be important for vascularization of implanted bone in humans [24].

It is still unclear what are the exact roles of various macrophage subsets in vascularization of biomaterials. To answer this question, Spiller et al. analysed the expression profiles of M1, M2a and M2c polarized macrophages *in vitro* with focus on the expression of genes and proteins important for angiogenesis. They also analysed the pro-angiogenetic potential of conditioned media from these polarized macrophage subsets in an *in vitro* sprouting assay with human endothelial cells. Interestingly, it was not one single subset of macrophages acting as the key regulator of vasculogenesis. Instead, an interplay between the M1 and M2 subset gave the best pro-angiogenic stimuli.

In order to confirm the findings from the *in vitro* studies, the vascularization process and the macrophage phenotype were evaluated *in vivo*. The following collagen scaffold types were subcutaneously implanted in a mouse model: glutaraldehyde-crosslinked (initiation of a moderate inflammatory response expected), soaked in LPS (M1 polarization expected) and unmodified (control). Ten days post-implantation, the scaffolds and the surrounding tissue were analysed with regards to the appearance of blood vessels (histological staining for CD31) and macrophage subsets in and around the scaffold (discrimination between M1 and M2). The unmodified scaffold showed no vascularization and was surrounded by a fibrous capsule in which was detected a strong signal for M2 macrophages. As expected, in the LPS soaked scaffold there were large numbers of inflammatory cells but no blood vessels, and were strongly stained for the M1 subset. In the third scaffold type, which was well vascularized, a staining for both, M1 and M2 macrophages was observed [24].

**Figure 7:**
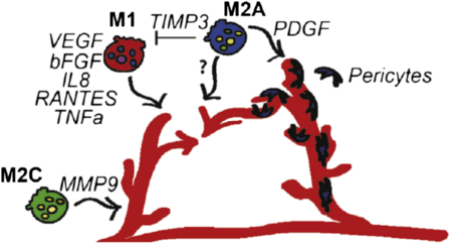
**Proposed model for macrophage-mediated angiogenesis.** M1 macrophages promote sprouting of blood vessels via secretion of VEGF, bFGF, IL8, RANTES, and TNFa. M2a macrophages promote fusion of blood vessels through as-yet unidentified secreted factors. M2a macrophages may also regulate the actions of M1 macrophages via production of TIMP3, and may recruit pericytes via secretion of PDGF-BB, although this was not directly assessed in this study. M2c macrophages may function in vascular remodeling, given their high levels of production of MMP9. Reproduced with permission from [28].

Taken together the findings of this study suggest that coordinated involvement of both subsets of macrophages guides new blood vessel formation (Figure 7). A model was proposed in which M1 macrophages promote sprouting of blood vessels via secretion of VEGF, bFGF, IL8, RANTES, and TNFalpha; M2c macrophages support angiogenesis, by increasing vascular remodeling via production of MMP9; M2a macrophages promote fusion of blood vessels through currently unidentified secreted factors. M2a macrophages may also regulate the actions of M1 macrophages via production of TIMP3, and may recruit pericytes via secretion of PDGF-BB. The interplay between M1 and M2 macrophages in regulating angiogenesis, and particularly the effects of timing requires much more work [24].

### Adaptive immunity

The adaptive (acquired) immune system is characterized by high antigen specificity. Main cell types of the adaptive immunity are B and T cells that recognize non self-proteins by their highly specified antigen receptors. The uniqueness of the adaptive immune system is the enormously high variety of these antigen receptors whereby a vast number of different antigens can be detected. Another important characteristic of the adaptive immune system is its memory. The memory enables a much faster reaction of this part of the immune system during a renewed contact with an already known antigen. Besides activation of the cells of the adaptive immune system via their receptors, another possible activation occurs via signals released by the innate immune system demonstrating the high interconnectivity between both parts of the vertebrate immune system.

In addition to its obvious crucial role in the fight against pathogens, the adaptive immunity also plays an important role during the healing process of a fractured bone, illustrating the interdependency between the bone and the immune system [37].

#### Negative impact of the immune system in the regenerative processes

After an injury, the disruption of the blood vessels leads to hematoma formation that is accompanied by inflammation. This immediate body reaction is a phylogenetically ancient, adaptive response [38]. In the context of wound healing, it is an indispensable step for the initiation of the healing cascade [4]. Wound healing is a multistage and complex process including a multitude of different regulatory mechanisms and molecules, especially in the inflammatory reaction, determining the course of regeneration versus scar formation.

**Figure 8:**
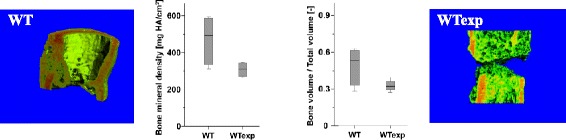
**Bone healing in mice under different housing conditions.** Analyzing bone healing in a mouse, which has been raised in a SPF (specific pathogen free) surrounding (WT) and a mouse, that had contact with pathogens and thus the possibility to develop its adaptive immune system (higher effector/ memory T cell count) (Wtexp): it became apparent, that the exposed animals showed a diminished healing capacity when compared with animals of SPF raising. This was documented through μCT evaluation showing lower bone mineral density and a lower bone volume/total volume in exposed animals. This data has been earlier published in the context of the negative influence of terminally differentiated CD8+ T cells in bone healing [13].

One of the key factors could be the age of the adaptive immune system. Whitby and Ferguson [39] showed that wounds in early mammalian embryos heal without scar formation [39]. At these early stages, the immune system is not yet fully developed. This leads to the assumption, that a fully developed immune system thought to be perfectly adapted to act against the invasion of pathogens, could have a negative impact in the course of wound healing (Figure 8). This raises the question whether the evolutionary development of such a highly efficient immune response takes into account drawbacks for the body’s regenerative capacity. The immune system ages throughout the life of an individual. Therefore, the cellular composition is different between a young and an older organism that has been exposed to a multitude of antigens and developed an immune memory. This aging process could be the reason for the diminished regenerative capacity of bone in the aged. This assumption is confirmed by the finding of an enhanced healing after rejuvenation of the immune system [40]. Furthermore, mice with a blunted immune system (germ free housing) develop a significant higher bone density compared to control animals [41].

The question is: Do certain cells of the adaptive immunity have a negative impact on wound healing? The answer is yes: CD8^+^ T cells as part of the adaptive immune response were found to negatively influence wound healing [42,43]. They could therefore be a promising therapeutic target in wound healing treatment strategies in general and especially in bone healing because the bone marrow microenvironment harbors, contrary to other tissues, a higher percentage of CD8^+^ T cells than CD4^+^ T cells under physiological conditions [44]. Reinke et al. has shown in humans that an enhanced level of terminally differentiated effector memory CD8 + T cells correlates with delayed fracture healing. This was further confirmed in a mouse osteotomy model where a specific depletion of CD8+ T cells led to an improved bone healing [13]. Whether the cells themselves or their secreted cytokines have to be finally targeted has still to be evaluated.

#### Positive impact of the adaptive immune system in regenerative processes

The T cell population is highly diverse. These cells secrete different inflammatory cytokines and proteins (like Wnt ligands) and thereby promote bone resorption and bone formation, respectively. Furthermore, bone homeostasis is regulated by T cells via their crosstalk with bone marrow stromal cells. Among the T cell population, CD4+ and CD8+ T cell subsets seem to play different roles in bone formation. *In vitro* studies showed that the osteogenetic differentiation in human MSC cultures significantly increases with conditioned media of unstimulated CD4+ T cells. This was further confirmed by the up-regulation of the expression of osteogenic markers such as runt-related transcription factor 2 (Runx2), osteocalcin (OC), bone sialoprotein (BSP), and alkaline phosphatase (ALP) [45]. In contrast, no such effect was seen with the conditioned medium from CD8^+^ cells. A positive role of CD4+ T cells was further evaluated in wound healing without identifying the responsible subset [42]. Regulatory T cells, a subpopulation of CD4+ T cells, are a promising candidate for a positive regulator in wound and bone healing. Several studies cement this assumption.

**Figure 9:**
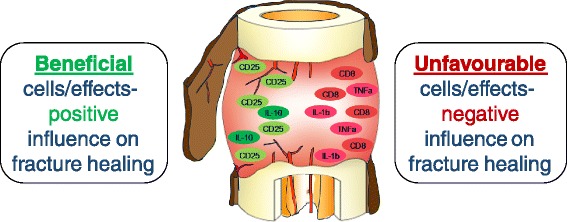
**Beneficial and unfavourable immune cells in bone healing.** The cellular composition of the bone hematoma that forms upon vessel disruption during injury, is an important criterion towards successful healing. To date certain cells of the adaptive immune system and their influence on regenerative processes have been determined which are depicted in this figure.

A correlation between high bone mass and decreased bone resorption was observed in mice with an increased amount of regulatory T cells [44,46]. In addition, regulatory T cells counteract transplant rejection due to their tolerogenic capacity [47], have a negative impact on osteoclasts [48] and were shown to enhance bone healing of a skull defect in mice when integrated in autologous bone graft [49] (Figure 9).

## Conclusion

The current therapies for the treatment of non-healing bone fractures and bone disease are still unsatisfactory. Fracture healing is a highly complex process involving different cells and released factors, tissue matrix and the inflammatory and immune system. For a successful healing outcome, the spatial and temporal interplay of these different components has to be well orchestrated. Therefore, in-depth understanding of bone regeneration on the molecular, cellular and tissue level is indispensable for the development of new therapeutic approaches.

Both the innate and adaptive immunity play a crucial role by initiating and regulating the healing cascades. The impact of immune cells in musculoskeletal diseases illustrates how crucial it is to better understand the signaling processes during the inflammatory phase of healing. This importance is further supported by the growing needs of our aging population, where the risk of fracture and poor recovery become more frequent. The aging population presents new challenges to both clinicians and researchers.

To develop new treatment strategies, the challenges we need to address include: (*i*) An early assessment of poor healing so that advanced treatment options can be implemented as soon as possible, for best outcomes, and (*ii*) Timely coordination of the therapeutic intervention with the progression of healing. There are already several approaches developed to address each of these aspects. Based on a better understanding of the signaling cascades and the types, roles and timing of presentation of the participating molecules and cells, a biomarker to “predict” the healing outcome of a patient would be most useful. In this context, the finding that a special subset of CD8+ T cells has a negative impact on fracture healing is already an important step in the early clinical evaluation of fractured patients.

The use of biomaterials to support the healing process is another important aspect in treating musculoskeletal disease. Biomaterials are used as a structural template for the regenerating tissues, including bone, and at the same time they can function as a source of biological factors. A biomaterial can be designed to optimize the regenerative mechanisms by delivering the right amounts of factors at the right time and to the right location within the bone healing area. The new generation of biomaterial scaffolds is being designed to recapitulate the local cytokine and/or growth factor milieu of development and remodeling, and to stimulate specific cell subsets to proliferate and differentiate in the desired and patient-specific way. One example discussed at the workshop was the incorporation of factors recruiting M1 macrophages over an initial period of time (to initiate vascularization) along with the factors (immobilized for sustained action) to recruit M2 macrophages (to mature and stabilize vasculature). So far, little is known about the specific role of the M1 and M2 types in the different musculoskeletal diseases and healing phases (e.g. revascularization of a fractured bone or a bone transplant) and further research is needed to evaluate their potential. This ORS workshop from 2014 with its topics of osteoimmunology in fracture healing, joint replacement loosening, and *in vitro* model systems demonstrates the wide spectrum of bone-immuno-interplay. Future research should pay attention to this relevant field.
